# *Sarcocystis* species in wild and domestic sheep (*Ovis ammon* and *Ovis aries*) from China

**DOI:** 10.1186/s12917-018-1712-9

**Published:** 2018-12-03

**Authors:** Hui Dong, Ruijing Su, Yinghua Wang, Zongxi Tong, Longxian Zhang, Yurong Yang, Junjie Hu

**Affiliations:** 1 College of Animal Science and Veterinary Medicine, Henan Agricultural University, Zhengzhou, 450002 China; 2 Center for Animal Disease Control and Prevention of Henan Province, ZhengZhou, 450002 China; 3 School of Biological Sciences, Yunnan University, Kunming, 650091 China

**Keywords:** *Sarcocystis*, Argali, Sheep, Parasite load, *S. tenella*, *S. arieticanis*, China

## Abstract

**Background:**

*Sarcocystis* species are intracellular protozoan parasites that can pose a threat to animal health and food safety. The aim of this study was to investigate the prevalence of infection with *Sarcocystis* infection in sheep from China.

**Results:**

In total, 52.51% (335/638) of tissue samples from domestic sheep contained sarcocysts through examination by light microscopy. The organisms were identified as *S. tenella* and *S. arieticanis* by molecular assays. Macroscopic *S. gigantea* and *S. medusiformis* were not found. The average sarcocysts loading was 18.07 ± 29.87 per square centimeter in the myocardium of domestic sheep. Furthermore, two specimens of argali (*Ovis ammon*) were examined and sarcocysts were found in the myocardium of one animal. According to the sequence of the *cox*1 gene of sarcocysts from argali, it was speculated as *S. tenella*.

**Conclusions:**

We found a high prevalence and parasite load of *Sarcocystis* in sheep from both central and northwest China. This report is the first to indicate that argali may be a natural intermediate host for *S. tenella*.

## Background

*Sarcocystis* are intracellular protozoan and food-borne parasites [1]. Currently, 196 valid *Sarcocystis* species are recognized [2], each of which has a strict host and genus specificity. The parasites form cysts that are found in the striated muscles and central nervous system of livestock, such as swine, cattle, and sheep [2, 3]. Human can be infected with *S. hominis, S. heydorni,* and *S. suihominis* by consumption of undercooked meat containing the sarcocysts [2, 4]. The European Food Safety Authority has classified *S. hominis* and *S. suihominis* as zoonotic hazards for which official meat inspections need to be vigilant [5]. Sarcocystosis contributes to weight loss, abortion, premature birth, and even death in sheep; these animals are usually infected by ingesting water and by foraging for feed contaminated with *Sarcocystis* sporocysts [2].

Most *Sarcocystis* species have an obligatory two-host life cycle, with carnivores as definitive hosts and herbivores as intermediate hosts [6]. There have been only four validated species described in sheep: the non-pathogenic macroscopic *S. gigantea* and *S. medusiformis* transmitted by felids and the pathogenic microscopic *S. tenella* and *S. arieticanis* transmitted by canids [2]. *S. tenella*, *S. arieticanis,* and *S. gigantea* are distributed worldwide, including China. However, *S. medusiformis* has only been reported from Italy, Iran, New Zealand, Spain, Jordan and Australia [2, 7].

In China, the annual production of meat from sheep in 2017 was 4851 thousand tonnes. The average prevalence of *Sarcocystis* infection in sheep was 41.52% (14,639/35254) in China [3], which was only slightly lower than that of the entire world (46.72%, 32,314/69158) [2]. *Sarcocystis* infection can cause economic losses in animal husbandry and represents a threat to public health and food safety [5]. However, there are few reports available in Chinese journals concerning sarcocystosis in sheep. In most studies, detection was done by naked eye and muscle squash. Further, studies of sarcocystosis in wildlife may provide a point of comparison for understanding the evolution and development of the parasite in domestic animals in human habitats. Argali (*Ovis ammon*) belongs to order Artiodactyla, suborder Ruminantia, infraorder Pecora, family Bovidae, subfamily Caprinae, tribe Caprini, genus *Ovis*. In the present work, we examined the prevalence, morphology, and molecular characteristics of *Sarcocystis* species in the muscles of domestic sheep and argalis from China.

## Results

Macroscopic sarcocysts were not found in 640 sheep hearts. The prevalence of infection with *Sarcocystis* species in the domestic sheep was 52.51% (335/638) (Table 1), and sarcocysts were detected in the myocardium of one argali out of two animals inspected by histological examination.

**Table 1:** Infection rate and loading of *Sarcocystis* species in domestic sheep

Batch number	Location^a^	City	Sample received date	No. of samples	No. of positives	Infection rate (%)	Density (cysts/cm^2^)
1	I	Zhengzhou	1 Mar 2014	155 hearts	20	12.90	6.35
2	II	Luoyang	18 Oct 2015	32 hearts	10	31.25	22.53
3	III	Xinxiang	20 Nov 2016	10 hearts	0	-	-
4	IV (n=254)	Jiaozuo	9 Nov 2015	70 hearts	62	93.70 (238/254)	26.96
5			25 Nov 2016	58 hearts	58		18.73
6			16 Oct 2017	62 hearts	55		19.73
7			19 Oct 2017	64 hearts	63		22.65
9	V (n=82)	Zhumadian	8 Oct 2015	15 hearts	3	10.98 (9/82)	0.56
10			30 Nov 2015	36 hearts	3		12.44
11			1 Dec 2015	20 hearts	2		0.33
12			7 Dec 2015	11 hearts	1		0.33
13	VI	Xinyang	8 Jul 2015	3 hearts	0	-	-
14	VII	Hami	4 Jan 2016	102 hearts	58	56.86	6.60
15	Total			638 hearts	335	52.51	18.07

^a^Sampling areas in Fig. 1.

**Fig. 1:**
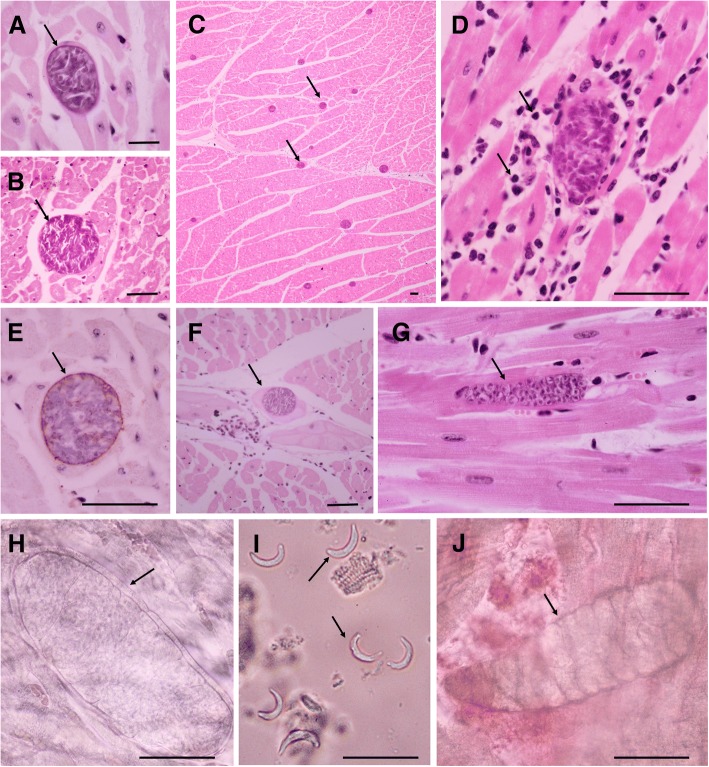
*Sarcocystis* infection in the myocardium of sheep. LM, H&E. Bar = 50 μm. **a** Thick-walled *S. tenella* sarcocyst, domestic sheep. **b** Thin-walled *S. arieticanis* sarcocyst, domestic sheep. **c** Numerous sarcocysts in sheep myocardium, domestic sheep. **d** Inflammatory cells infiltrating the area around a sarcocyst, domestic sheep. **e** Cysts testing negative for *Toxoplasma gondii*, domestic sheep. **f** A sarcocyst in Purkinje fibers, domestic sheep. **g** Immature sarcocyst with numerous metrocytes, domestic sheep. **h** Bradyzoites are located in the numerous chambers, created by the septa of the sarcocyst; the finger-like villar protrusions around a cyst wall; myocardium squash, unstained, domestic sheep. **i** Bradyzoites in pepsin-digestion liquid; unstained, domestic sheep. **j** Sarcocyst from myocardium squash, unstained, argali

Thick-walled cysts of *S. tenella* and thin-walled cysts of *S. arieticanis* were observed in the samples by light microscopy (Fig. 1a, b). The proportion of thick-walled sarcocysts was higher than that of thin-walled sarcocysts (*P* < 0.05). The average sarcocyst loading was 18.07 ± 29.87 per square centimeter; the tissue with the highest number of sarcocysts observed (*n* = 302) is shown in Fig. 1c. Compared to the fraction of samples with less than 10 sarcocysts/cm^2^ was 201/335 (60.00%); 10–50 sarcocysts/cm^2^ were present in 95/335 (28.36%) of the samples (*P* < 0.01); and more than 50 sarcocysts/cm^2^ were present in 39/335 (11.64%) of the samples (*P* < 0.01). We observed inflammatory cells around the sarcocysts, indicating myositis, in 3.45% (22/638) of the samples (Fig. 1d).

Under light microscopy, the sarcocysts appeared fusiform or oval-shaped; they were not *Toxoplasma gondii* cysts, which confirmed by IHC-staining (Fig. 1e). Sarcocysts were also observed in Purkinje fibers (Fig. 1f). Immature sarcocysts with numerous metrocytes were found in the myocardium from domestic sheep (Fig. 1g). The size of *S. arieticanis* cysts was 6.51–105.00 μm × 6.46–37.63 μm (*n* = 40); villar protrusions were hair-like; the walls of the cysts were 0.24 ± 0.23 μm thick, and the size of the bradyzoites was 2.68–6.27 μm × 0.9–1.89 μm (*n* = 20). For *S. tenella,* the size of cysts was 15.20–88.00 μm × 11.19–33.11 μm (n = 40); the villar protrusions were finger-like (Fig. 1h); the wall was 0.56 ± 0.54 μm thick, and bradyzoites measured 3.56–5.62 μm × 1.21–1.84 μm (*n* = 20). The sarcocysts contained crescent-shaped bradyzoites located in numerous chambers formed by septa (Fig. 1h, i). We also found sarcocysts in the myocardium of argali: the size of the cysts was 16.74–17.83 μm × 55.23–61.74 μm (*n* = 3); the villar protrusions were finger-like, and the wall was 0.13 ± 0.09 μm thick (Fig. 1j). The morphology of sarcocysts found in argali mostly resembled that of the *S. tenella* cysts in domestic sheep.

Variation associated with the risk factors of location and season of the year is shown in Table 2. The locations of the provinces where the samples were collected showed no correlation with *Sarcocystis* prevalence (*P* > 0.05). The risk of *Sarcocystis* infection in the autumn was higher than that in the spring and winter (*P* < 0.01).

**Table 2:** Odds ratio of province and season as risk factors for the prevalence of infection with *Sarcocystis* species in sheep

Factor	Category	Positive/examined (%)	OR	95% Cl	*P* value
Province	Henan	277/536 (51.68)	-	-	-
	Xinjiang	58/102 (56.86)	1.233	0.8042-1.889	*P* = 0.3870
Season*	Spring	20/155 (12.90)	-	-	-
	Winter	61/133 (45.86)	5.719	3.200-10.22	*P* < 0.0001
	Autumn	254/347 (73.20)	3.224	2.127-4.886	*P* < 0.0001

OR Odds ratio;

“*” indicates significant difference.

**Fig. 2:**
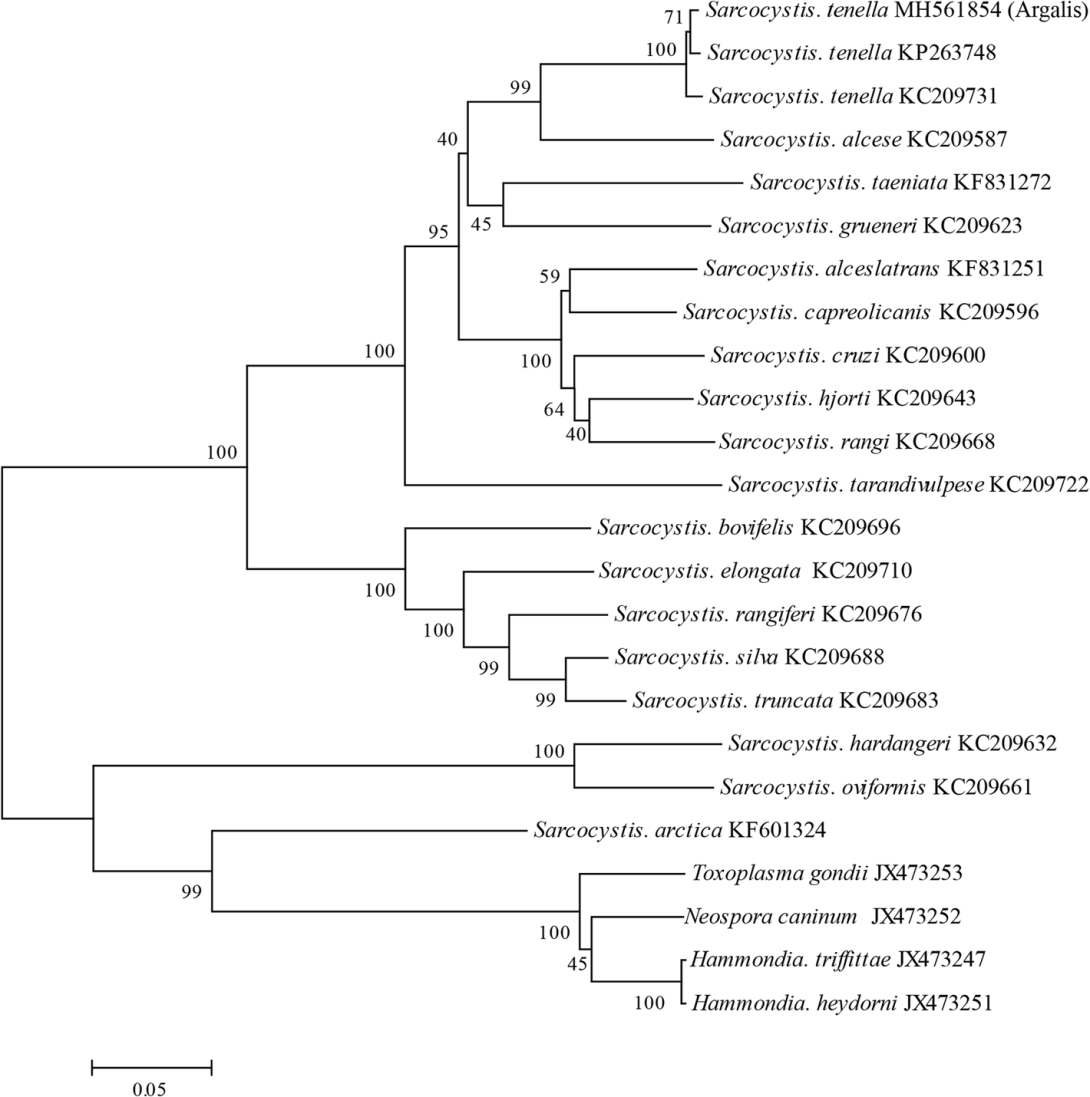
Phylogenetic tree among the *Sarcocystis tenella* from argali identified in this study and other *Sarcocystis* spp. The phylogeny was inferred from neighbor-joining analysis of the mitochondrial *cox1* sequences based on distances calculated using the Kimura two-parameter model. Bootstrap values *N* > 50% from 1000 replicates are shown at the nodes. *Sarcocystis spp.* (MH561854) is closely related to *S. tenella* (KP263748 and KC209731), indicating that argalis maybe intermediate hosts for *S. tenella*

The *cox1* amplification and sequence analysis verified that sarcocysts from argali were *S. tenella* (Fig. 2)*.* Furthermore, the mitochondrial *cox1* nucleotide sequences from sarcocysts of argali were submitted to GenBank (accession number MH561854). The sequence was approximately 989 bp in length and had high identity with *S. tenalla*. The sequence showed 99% identity with those of *S. tenalla* (KC209723-KC209732) (KP263746-KP263751) from Norwegian sheep and Polish Tatra chamois, respectively. Additionally, there was 97% identity with *S. tenalla* (MF039322 and MF039323) from sheep, 93% identity with *S. capracanis* (KU820974 and KU820977) from goats, and 90% identity with *S. heydorni* (KX057994 and KX057995) from cattle in China. The phylogenetic analysis based on near full-length mitochondrial *cox1* sequences placed the sequence of *S. tenella* within a clade comprising the sequence of *S. tenella* (KP263748 and KC209731).

## Discussion

The determination of the prevalence value for sarcocystosis in sheep depends on the method of detection. The method of using squashed tissue is fast and simple; however, the sensitivity is inferior to methods using tissue sectioning and PCR [2]. Usually, the detection ratio of *Sarcocystis* infection can be improved two-fold in swine by examining histological sections rather than squashed muscle [8]. Bradyzoites have been detected in pepsin-digested samples that had previously been found free of cysts using the squashed muscle method. The pepsin digestion method combined with PCR assays may be more sensitive than other methods for detecting *Sarcocystis* species [8]. Therefore, the 52.51% prevalence of *Sarcocystis* infection found in this study of domestic sheep may be lower than the real value.

Sarcocysts have been reported in the diaphragm, esophagus, heart, and skeletal muscles of domestic sheep and goats in China. Most reports of *Sarcocystis* in sheep have been published in Chinese. In this study, the prevalence of sarcocystosis was higher than the national average in China (41.52%) [3]. It was also higher than the corresponding values from Asia (48.02%, 26,061/54270), Europe (31.42%, 3158/10051), and South America (36.22%, 343/947), but lower than the prevalence of sarcocystosis in Oceania (93.31%, 990/1061), Africa (54.67%, 621/1136), and North America (67.40%, 1141/1693) [2, 3]. In this study, the prevalence of sarcocysts in sheep was high (52.51%), but the prevalence of myositis was low (3.45%). The results indicate that in most cases, the sarcocysts are non-pathogenic for the sheep.

*S. gigantea* and *S. medusiformis* are two species with macroscopic cysts [2]. *S. gigantea* has not been found in the heart but occurs primarily in the esophagus, larynx and tongue muscles [9]. *S. medusiformis* cysts are found primarily in the diaphragm, abdominal muscles, and carcasses [2]. We did not find macroscopic sarcocysts in any of the sheep heart samples examined; this is in accordance with the distribution of *Sarcocystis* in host tissues described by Dubey et al. [2]. In our study, the sarcocysts in heart muscles were identified as cysts of *S. tenella* and *S. arieticanis* by morphology, location, host, and molecular characteristics. The average sarcocysts loading in muscle from domestic sheep was 18.07 ± 29.87 cysts per square centimeter, which is much higher than that in beef (1.40 cysts/cm^2^) and feral pigs (3.03 cysts/cm^2^) from the U.S. and mutton (7.84/cm^2^) from China [8, 10, 11]. The higher parasite load indicates that the domestic sheep we examined had contact with an environment severely contaminated with sporocysts or the specific strains. Although most *Sarcocystis* species are non-pathogenic for sheep, infection may have an adverse impact on the health of domestic sheep, may endanger meat quality, and may lead to economic losses for the livestock industry.

Both Henan Province (located in central China) and Xinjiang Province (located in the northwest of China) had a high prevalence of *Sarcocystis* infection, indicating that *Sarcocystis* may be widespread in China. Usually, sarcocysts appear in striated muscle 1~2 month post-ingestion of sporocysts. The risk of *Sarcocystis* infection in the autumn was higher than that in winter or spring (*P* < 0.01) (Table 2). It has been speculated that summer and autumn may pose a higher risk for infection with *Sarcocystis* sporocysts compared with winter or spring. Few epidemiological studies have assessed the risk factors associated with *Sarcocystis* in sheep. However, it has been hypothesized that contact with canids or felids, a moist environment, and age are risk factors for *Sarcocystis* [2]*.*

*Sarcocystis* species have strict host-genus specificity, and they infect many wild and domestic ruminant animals [2]. *S. ferovis* was found in Bighorn sheep, but it was not transmissible to domestic sheep [12]. However, *S. cruzi* was present in both domestic cattle and wood bison [13]. A *Sarcocystis* species was first identified from wild sheep (*Ovis musimon*) [14], and *S. tenella* and *S. arieticanis* were later identified from European Mouflon (*Ovis ammon musimon*) [15]. In this study, only microscopic sarcocysts were found in argali.

The 18S rRNA gene, the 28S rRNA gene and the internal transcribed spacer 1 (*ITS1*) were employed to identify the molecular characteristics of *Sarcocystis* spp. Gjerde reported that *cox1* is a useful genetic marker for Sarcocystidae [16], because it is helpful to resolve the unclear species boundaries of closely related *Sarcocystis* spp. in different hosts. Hu et al. reported that the 18S rRNA, 28S rRNA, and *cox1* genes for *S. tenella* shared high identities with those of *S. capracanis*, i.e., 99.0, 98.3, and 93.6%, respectively [17, 18]. *Cox1* seemed to perform better than 18S rRNA or 28S rRNA for distinguishing *S. tenella* from *S. capracanis* [16]. The sequence of sarcocysts from argali (MH561854) was submitted to GenBank. The phylogenetic analysis based on near full-length mitochondrial *cox1* sequences placed the sequence of *S. tenella* within a clade including the sequences of *S. tenella* (KP263748 and KC209731) (Fig. 2). This work is the first report concerning the *Sarcocystis* infection in argali from China, indicating that argali may be a natural intermediate host for *S. tenella*.

## Conclusion

We found a high prevalence and parasite load of *Sarcocystis* in sheep from both central and northwestern China. We also examined two specimens of argali (*Ovis ammon*) and found sarcocysts in the myocardium of one animal. The sequence of the *cox*1 gene of sarcocysts from argali suggested that the species was *S. tenella*. This report is the first to indicate that argali may be a natural intermediate host for *S. tenella*.

## Methods

### Sampling of naturally infected animals

**Fig. 3:**
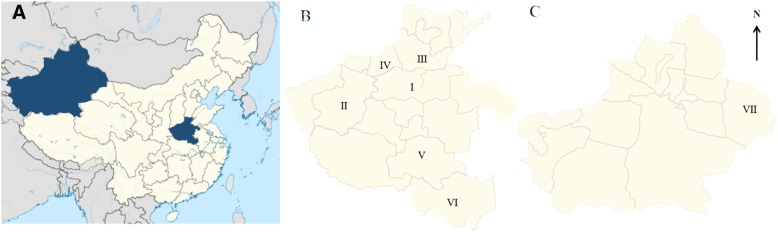
Map showing the location of samples received from Henan and Xinjiang provinces in China. Map adapted from Google Earth. **a** Location of Henan and Xinjiang provinces in China; **b** Areas of collection within Henan Province. I: Zhengzhou, II: Luoyang, III: Xinxiang, IV: Jiaozuo, V: Zhumadian, VI: Xinyang; **c** Area of collection within the Xinjiang Uygur Autonomous Region. VII: Hami

From March 2014 to October 2017, we examined a total of 638 domestic sheep hearts (all animals over six months old) from selected areas in Henan and Xinjiang provinces for *Sarcocystis* infection (Fig. 3, Table 1). We also evaluated samples from the hearts and diaphragms of two argalis (males, 2–3 years old) for the occurrence of sarcocysts. The argalis came from zoos in 2017 and 2018, where they had died from diarrhea. All samples were transported to the Laboratory of Veterinary Pathology, Henan Agricultural University (Zhengzhou, Henan, China) in cooler boxes. All samples were examined for macroscopic and microscopic cysts of *Sarcocystis*.

### Microscopic examination

Muscles were cut from each sheep heart and squashed between two glass slides, then examined microscopically at × 10 magnification for the presence of sarcocysts. Three pieces of muscle from each sample were fixed in 10% formalin in a neutral buffer for paraffin sections (5 μm) and were stained with H&E (hematoxylin-eosin) and IHC (immunohistochemistry) reagents. Rabbit anti-*Toxoplasma gondii* polyclonal antibody was kindly provided by Dr. Dubey (ARS, USDA). A mouse- and rabbit-specific 3, 3′-diaminobenzidine- horseradish peroxidase (HRP/DAB) Avidin-biotin complex (ABC) IHC detection kit was purchased from Abcam (ab64264). The sarcocysts were observed and photographed under a Leica DFC320 light microscope.

### Pepsin digestion examination

Individual samples of the myocardium (50 g) were homogenized and digested in acidic pepsin [19]. The digested tissues were examined for bradyzoites and cysts of *Sarcocystis* species by light microscopy.

### Molecular identification

Individual sarcocysts were isolated from the muscle of sheep and argalis under a stereomicroscope. DNA was extracted from single sarcocysts using a commercial DNA extraction kit (Tiangen Biotec Company, DP304, China). PCR was performed to amplify a segment of the mitochondrial cytochrome c oxidase subunit 1 gene (*cox1*) using the specific primer pairs of SF1 and SR9. The amplified PCR product was approximately 1038 bp [16, 18, 20]. The amplified PCR products of sarcocysts were sent to Beijing Nuosai Biological Engineering Biotechnology Company for bi-directional sequencing on an ABI PRISM™ 3730 XL DNA Analyzer using the BigDye Terminator v3.1 Cycle Sequencing Kit (Applied Biosystems, Foster City, CA, USA). The obtained sequences were analyzed by BLAST. MEGA 6.0 software and was used to construct a phylogenetic tree of the *S. tenella* isolates using the neighbor-joining method (Kimura two-parameter model). Bootstrap analysis using 1000 replicates was used to assess the robustness of clusters.

### Statistical analysis

Statistical analysis was performed with the GraphPad Prism 4.0 software (GraphPad Software Inc., San Diego, CA, USA). The data were analyzed by a Chi-square test or Fisher’s exact test to determine the association between infection with *Sarcocystis* and risk factors such as location (Xinjiang and Henan provinces) and seasons (spring, autumn, and winter). *P* < 0.05 was considered statistically significant.

## Abbreviations

*cox1*: Mitochondrial cytochrome c oxidase subunit 1 gene
H&E: Hematoxylin and eosin staining
IHC: Immunohistochemistry
PCR: Polymerase chain reaction
